# An insulin growth factor-I/II-neutralizing monoclonal antibody in combination with epidermal growth factor receptor inhibitors potently inhibits tumor cell growth

**DOI:** 10.7150/jca.69064

**Published:** 2022-03-21

**Authors:** Guofang Ma, Chengyue Tan, Yaming Shan, Ningyi Shao, Feng Wang, Dimiter S. Dimitrov, Liping Wang, Qi Zhao

**Affiliations:** 1 Key Laboratory for Molecular Enzymology and Engineering, Ministry of Education, School of Life Sciences; Jilin University, Changchun, Jilin, China.; 2 Engineering Laboratory for AIDS Vaccine, Jilin Universtiy, Changchun Jilin, China.; 3 Cancer Centre, Faculty of Health Sciences, University of Macau, Taipa, Macau, China.; 4 MoE Frontiers Science Center for Precision Oncology, University of Macau, Taipa, Macau SAR, China.; 5 Key Laboratory of Molecular Medicine and Biotherapy, School of Life Science, Beijing Institute of Technology, Beijing, China.; 6 Department of Medicine, Center for Antibody Therapeutics, University of Pittsburgh, Pittsburgh, Pennsylvania, United States.

**Keywords:** IGF-I, IGF-II, m708.5, monoclonal antibody, EGFR, gefitinib

## Abstract

The insulin-like growth factors (IGFs), IGF-1 and IGF-II, which bind to the IGF receptor type 1 (IGF-1R) and the insulin receptor (IR), have been implicated in the growth, survival, and metastasis of tumor cells. We have previously identified a novel human monoclonal antibody (mAb), m708.5, which neutralizes both human IGF-I and IGF-II, and potently inhibits phosphorylation of the IGF-1R and the IR in breast cancer cells. In this study, m708.5 exhibited very strong synergy with the epidermal growth factor receptor (EGFR) inhibitor gefitinib, and synergy with chemotherapeutic agents *in vitro* against either neuroblastoma or breast cancer cells. In xenografted models, the combination of m708.5 and gefitinib significantly inhibited LAN-1 cell growth better than single agent alone. Taken together, these results support the clinical development of m708.5 for solid tumors with potential for synergy with chemotherapy and EGFR inhibitors.

## Introduction

The insulin-like growth factor (IGF) family contains IGF-I and IGF-II, IGF receptor type 1 (IGF-1R), IGF receptor type 2 (IGF-2R), and six IGF binding proteins (IGFBPs) 1. The central components, IGF-1R and its ligands (IGF-I and IGF-II), have been implicated in the control of cellular proliferation and survival and of organism growth 2. IGFs, IGF-I and IGF-II, consist of 70 amino acids and 67 amino acids, respectively. They share 62% homology in protein sequence. IGFs display about 50% homology with insulin 3, 4. Upon IGF-I or IGF-II binding, the intrinsic tyrosine kinase activity of IGF-1R is switched on to induce IGF-1R autophosphorylation. Insulin receptor substrate 1 (IRS-1) is subsequently phosphorylated as it is recruited to IGF-1R. The activated IRS-1 triggers PI3K/Akt/mTOR pathway that blocks apoptosis. Besides, IGF-1R activates the SH2 containing protein (SHC), which initiates Ras/Raf/MAPK signaling cascade, resulting in cell proliferation 5, 6. Two isoforms of insulin receptor (IR), IR-A and IR-B, are translated from different splicings of IR mRNA. IR-A has high affinity to IGF-II, and it is activated by insulin and IGF-II to promote survival, motility and invasiveness of cancer cells 7, 8.

IGFs are produced in most tissues, and exert mitogenic effects in cell proliferation, survival and metabolism through endocrine, autocrine and paracrine mechanisms 9. IGFs are key mediators in mammary gland function and maintenance. Abnormal expression of IGF-II and/or its receptors is also reported in a wide range of cancers including breast, lung, colorectal, thyroid, bladder, primary liver and various sarcomas. Activation of the IGF-1R and IR by IGF-II has been shown to be the predominant IGF signaling receptor in different cancer 10, 11.

Most of monoclonal antibodies (mAbs) targeting IGF axis have been focused on targeting IGF-1R. Anti-IGF-1R mAbs can block IGFs binding to IGF-1R, induce IGF-1R internalization and degradation. However, therapeutic results of these mAbs are disappointing 12-14. Anti-IGF-1R mAbs do not inhibit the hybrid receptor IGF-1R/IR-A mediated signaling. Inhibition of IGF-1R signaling can also be bypassed by IGF-II dependent IR-A activation 15, 16. Several types of antibodies, including mAbs, bispecific antibodies, and antibody fragments, against IGF-II have been reported 15-18, which markedly deactivate the IGF-1R/IR and affect tumor growth. Previously, we reported that a human mAb m708.5, cross-reactive to IGF-I and IGF-II, potently inhibited IGF-induced IGF-1R signaling as well as suppressed phosophorylation of these receptors. Our results demonstrate that m708.5 has more potential inhibition effects on tumor cell growth 19. The IGF signaling activates PI3K/Akt pathway that initiates activation of mTOR, resulting in increased protein synthesis and cell mitosis that favor tumor growth 20. Combination therapy with m708.5 and a mTOR inhibitor, temsirolimus, significantly inhibits neruoblastoma xenograft growth and enhances mouse survival 21.

Some studies indicate that epidermal growth factor receptor (EGFR) cross-talks with IGF-1R in a wide range of epithelial tumours 22, 23. IGF-1R signal pathway is highly associated with resistance to the EGFR inhibitors in breast cancer cells. In this study, we further investigate the anti-IGF-I/II IgG1 m708.5 against neuroblastoma and breast cancer cell lines as well as xenografts models alone and in combination with the EGFR inhibitors. These studies will provide the rationale and preclinical data to guide the treatment of EGFR inhibitor gefitinib.

## Materials and methods

### Neuroblastoma and breast cancer cell lines

Neuroblastoma cell lines (LAN-1) was provided by RIKEN. Breast cancer cell lines (BT474, MCF-7, SK-BR-3, MD-MB-231, T47D) were provided by Cell Bank/Stem Cell Bank, Chinese Academy of Sciences. BT474 cells were maintained in RPMI1640 (GIBCO) supplemented with 10% heat-inactivated fetal bovine serum (FBS) (GIBCO). MCF-7, SK-BR-3, MD-MB-231 and T47D cells were maintained in DMEM (GIBCO) supplemented with 10% heat-inactivated FBS.

### Drugs

Gefitinib, cisplatin and pimasertib were purchased from DC Chemicals. These drugs were dissolved in dimethylsulphoxide (DMSO) as the stocks and freshly diluted in RPMI1640 or DMEM medium for the experiments. Other chemicals were purchased from Sigma-aldrich.

### Antibody expression and purification

The soluble IgG1 protein of m708.5 was expressed by the stable CHO-s cell line as previously described 21. The stable CHO-s cells were maintained in serum-free Opticho serum free medium (GIBCO). After 4-day expression, the culture supernatant was collected and purified using the MabSelect affinity chromatograph medium (GE Healthcare). The purified IgG1 was subsequently concentrated using an Amico® Ultra-15 10K centrifugal filter device (Merk Millipore). The purity of m708.5 IgG1 was analyzed by SDS-polyacrylamide gel electrophoresis (SDS-PAGE) and size-exclusion chromatography (SEC).

### Enzyme-linked immunosorbent assay (ELISA)

The 50 ng of human IGF-I (hIGF-I), IGF-II (hIGF-II) (PeproTech) per well were coated on 96-well ELISA plates overnight at 4 °C. Next day the serial dilutions of IgG1 m708.5 were incubated with antigens for 1 h at RT. Bound IgG1s were detected with horseradish peroxidase (HRP) conjugated anti-human Fc antibody (Jackson ImmunoResearch). The 20% H_2_SO_4_ was added and the reaction was read at 450 nm.

### Flow cytometric analysis

Rabbit anti-IGF-1R antibody (Cell Signaling) was incubated with tumor cells for 30 min on ice. Then, tumor cells were incubated with the secondary fluorescein Isothiocyanate (FITC)-conjugated anti-rabbit IgG antibody (Bio-Rad). The detection was performed using a BD Bioscience C6 flow cytometer.

### Cell proliferation assay

Tumor cells were seeded into tissue culture 96-well plates in cell culture medium containing 2.5% FBS overnight. Tumor cells were treated with the mixtures of IgG1 m708.5 and drugs, or drugs alone at different concentrations. After 48 h, cell viability was determined using an MTS assay kit by reading at 450 nm. The experiments were repeated 2-3 times. The calculated results were the percentage of cell growth relative to the control cell growth. Dose response curves were constructed for each drug and the combination at a fixed molar ratio (defined as the ratio of the two drugs at their maximum effective dose). Multi drug effect analysis was used to calculate the interaction between the drug and combination as previously described 21.

### *In vivo* therapy in xenografted models

All animal studies were conducted using protocols approved by the Animal Ethics Committees, University of Macau. Six to eight-week-old female nude mice were provided by the Animal Facility at Faculty of Health Sciences. Mice were randomly divided into groups (n=5 per group). Tumor xenografts were established by subcutaneous (s.c.) implantation of 1×10^6^ LAN-1 cells. When the average tumor volume reached 100mm^3^, mice were treated with either PBS (i.v., twice weekly for 3 weeks), 0.1 mg m708.5 (i.v., twice weekly for 3 weeks), 0.05mg or 0.125mg gefitinib (i.p., five times weekly for 3 weeks) or both m708.5 and gefitinib. The tumor volume was calculated by vernier caliper twice a week after inoculation. Tumor volume (mm^3^) was calculated by: [length (mm) × width (mm)^2^]/2. Tumor growth inhibition (TGI) was calculated as (1 - *T*/*C*) × 100, where *T* is the final tumor volumes from a treated group and *C* is the final tumor volumes from the control group. Statistical significance was determined using two ways ANOVA. P values are represented as: * *p* < 0.05, ** *p* < 0.01, *** *p* < 0.001, and **** *p* < 0.0001.

### RNA sequencing and analysis

Tumor samples collected from treated and non-treated xenografted mice were chosen for RNA extraction. Briefly, the samples were ground in liquid nitrogen, separately. RNA was extracted from tumor samples using Trizol reagent (Invitrogen) according to the manufacturer's instructions. The raw reads from the RNA-seq were aligned by Hisat2 with default parameters to hg19 24, and the gene expressions were extracted by HT-seq 25. The differentially expressed genes were detected by DESeq 26.The gene set enrichment analysis (GSEA) was conducted by the Bioconductor package ClusterProfiler (https://bioconductor.org/packages/release/bioc/html/clusterProfiler.html) 27.

## Results

### Characterization of anti-IGF-I/IGF-II IgG1 m708.5

**Figure 1:**
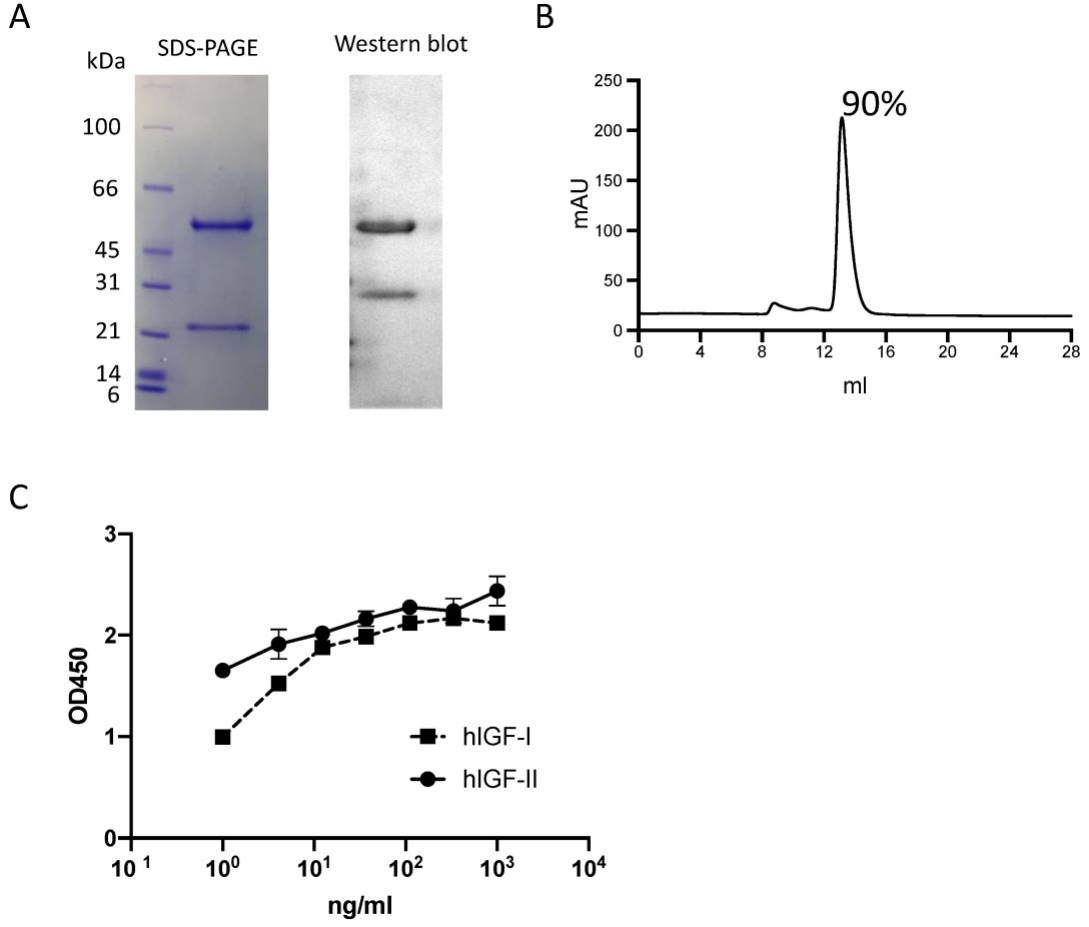
** Characterization of IgG1 m708.5. A.** Purity of purified IgG1 m708.5 by SDS-PAGE and Western blot. **B.** Purity of m708.5 by the size-exclusion column. **C.** Binding of IgG1 m708.5 to hIGF-I and hIGF-II by ELISA. IgG1 m708.5 were serially diluted and added to wells coated with IGF-I or IGF-II. Bound IgG1 was detected with an HRP-conjugated anti-human Fc antibody and optical densities (O.D.) measured at 450 nm.

The m708.5 IgG1 was expressed and purified. The purity of IgG1 protein was analyzed by SDS-PAGE (**Fig. 1A),** Western blot (**Fig. 1A**) and SEC (<10% aggregates) (**Fig. 1B**). Its binding abilities were confirmed by ELISA assays. As the results (**Fig. 1C**), m708.5 showed the cross-reactivity to both human IGF-I and IGF-II antigens.

### Expression of IGF-1R on tumor cell lines

**Figure 2:**
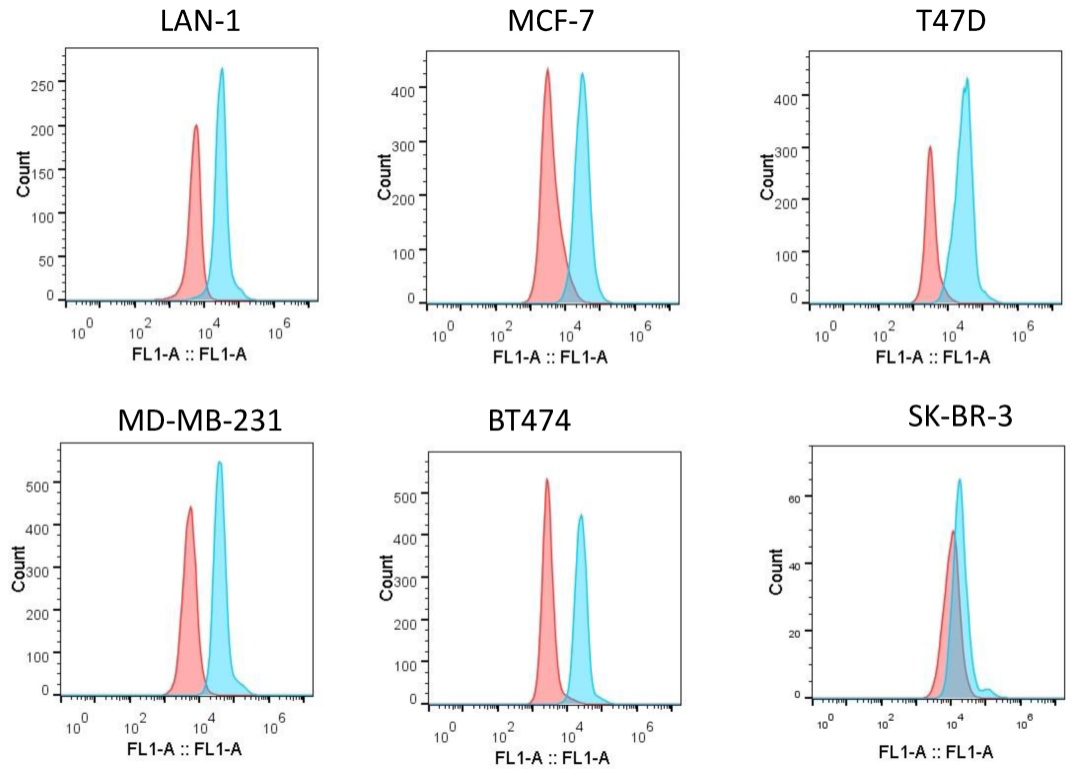
** Expression of IGF-1R on tumor cell lines.** Flow cytometric analysis of IGF-1R expression on the surface of different tumor cell lines was detected with rabbit anti-IGF-1R antibodies, followed by the FITC-conjugated anti-rabbit antibodies as the secondary antibody.

Increased expression of IGF-1R was a compensatory survival mechanism in pediatric cancer cells 28. Our previous results have shown that IGF-1R was overexpressed on neuroblastoma cells 21. We tested whether *in vitro* m708.5 sensitivity could be correlated with IGF-1R in breast cancer cells. We used anti-IGF-1R antibodies to assay for receptor expression on cell surface of breast cancer cells (MCF-7, MD-MB-231, T47D, SK-BR-3 and BT474) and neuroblastoma cells (LAN-1) by flow cytometry. All breast cancer cells, including Her2+ and triple negative cells, were observed to exhibit high expression of IGF-1R (**Fig. 2**). Thus, it appears that IGF-1R receptors are important for either breast or neuroblastoma cancer cell growth and as potential targets for the antibody m708.5.

### Antiproliferative activity of m708.5 in combination with chemodrugs against tumor cell lines *in vitro*

**Figure 3:**
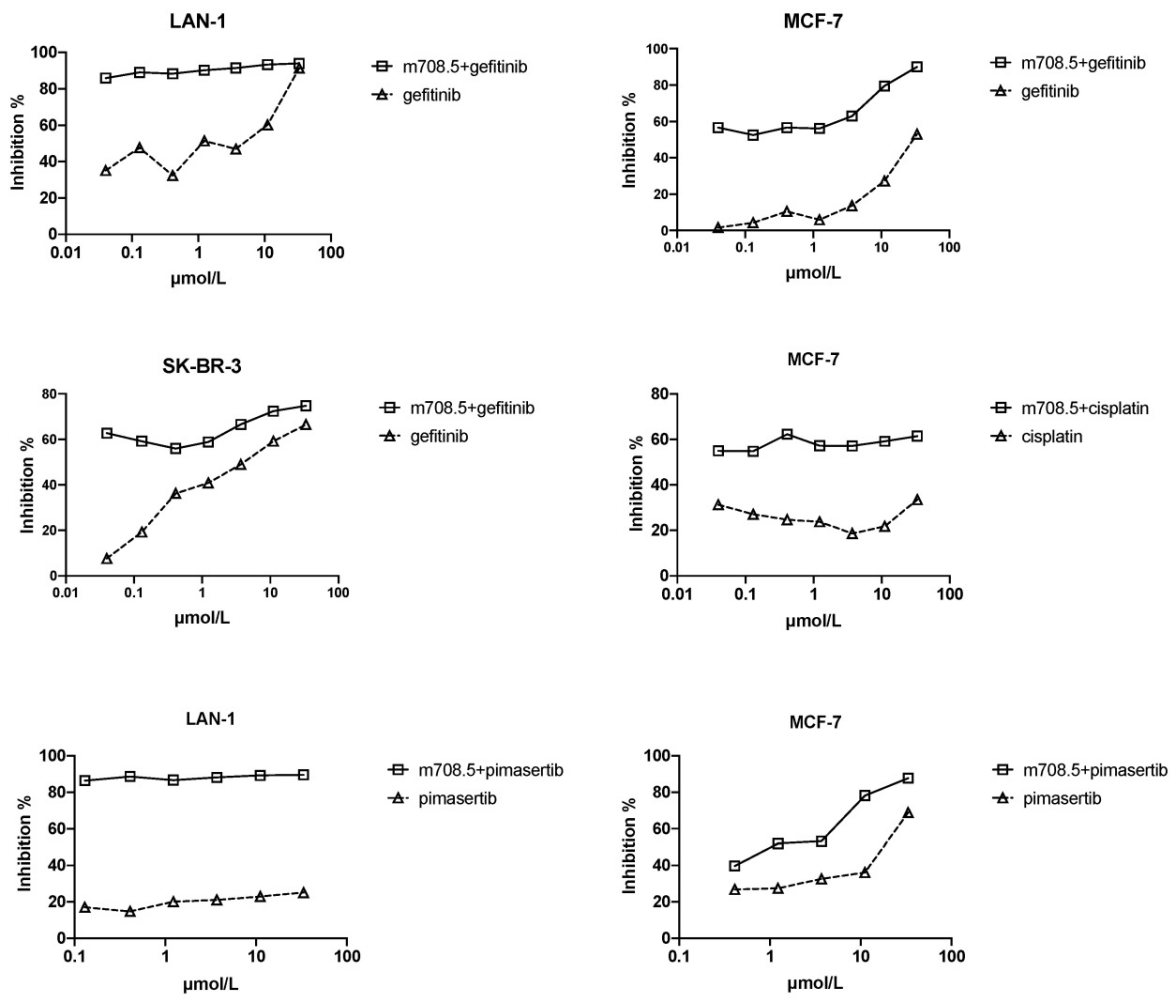
**Antiproliferative activity of m708.5 in combination with chemodrugs in tumor cell lines.** Cell proliferation of LAN-1, MCF-7 and SK-BR-3 cell lines cultured in 2.5% FCS-conditioned medium after 48-hr exposure to different drugs alone or in combination with m708.5 IgG1 at drug concentration indicated in figures. Numbers of viable cells were determined using MTS method and expressed as the percentage of inhibition on the *y-*axis.

Gefitinib (EGFR inhibitor) demonstrates relative safety and clinical activity in the treatment of multiple solid tumors. It was tested in combination with m708.5 because IGF-1R as an escape pathway mediates resistance to EGFR inhibitors 29. To test whether m708.5 could enhance chemotherapy, it was tested in combination with several representative drugs used in the treatment of multiple tumor cell lines, including gefitinib, pimasertib and cisplatin (**Fig. 3**). As shown in **Table 1**, for neuroblastoma cell line LAN-1, which is widely used in drug studies, the chemodrugs plus m708.5 exhibited increased antiproliferative activity. Strong synergism was seen with mean combination index values (CI) (gefitinib=0.012, and pimasertib=0.002). For two breast cancer cell lines (MCF-7 and SK-BR-3), gefitinib combination shows synergistic effect with CI =0.056 for MCF-7 and 0.14 for SK-BR-3, whereas pimasertib showed synergistic effect for MCF-7 with CI=0.03. Remarkably, the combination of m708.5 with the EGFR inhibitor gefitinib yielded the synergistic effects in all cell lines. These results suggest m708.5 enhances antitumor efficacy in combination with gefitinib against either neuroblastoma or breast cancer cells.

### Inhibition of tumor cell growth by m708.5 in combination with gefitinib *in vivo*

**Figure 4:**
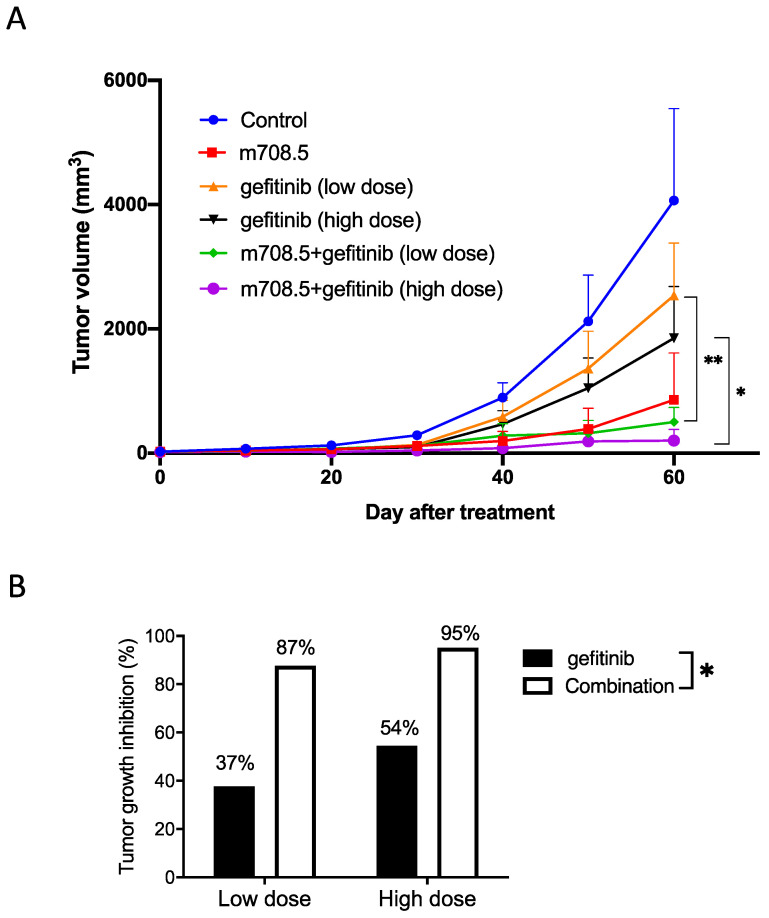
** Therapy of neuroblastoma xenografts with m708.5 combined with gefitinib. A.** Mice were inoculated s.c. with LAN-1 neuroblastoma cells. Tumor-bearing mice were assigned to treatment with either 0.1 mg m708.5 (i.v., twice weekly for 3 weeks), 0.05 mg or 0.125 mg gefitinib (i.p., 5 times weekly for 3 weeks) or both m708.5 and gefitinib. **B**. Tumor growth regression was calculated on Days 60 after treatment in (A).

Antitumor activity of m708.5 in combination with gefitinib was evaluated *in vivo* against LAN-1 cell line when grown as xenografts in nude mice. Mice received treatment with either 0.1 mg m708.5 (i.v., 3 weeks), low-dose 0.05 mg or high dose 0.125 gefitinib (i.p., 3 weeks) or combination of m708.5 and gefitinib. As shown in **Fig. 4A,** it was observed that both gefitinib and m708.5 were highly active in LAN-1 xenografted models when administered as a single agent. Tumors in the control grew with a time to reach 1000 mm^3^ of 40 days, whereas gefitinib (high dose)-treated and m708.5-treated tumors reached the same volume after 50 and 60 days, respectively, indicating a significant growth delay. Compared with single gefitinib treatment, there was significant tumor regression with the combination treatments of gefitinib and m708.5. On day 60 after treatment, gefitinib alone inhibited tumor growth by 37% at low dose and 54% at high dose. However, when gefitinib was combined with m708.5, inhibition increased to 87% at low dose and 95% at high dose (**Fig. 4B**). These results, which are consistent with those observed *in vitro*, demonstrated that the combination therapy of EGFR inhibitors and IGF-I/II-neutralizing antibodies had substantially better antitumor efficacy than individual treatment.

### Transcriptome changes of m708.5 in combination with gefitinib *in vivo*

**Figure 5:**
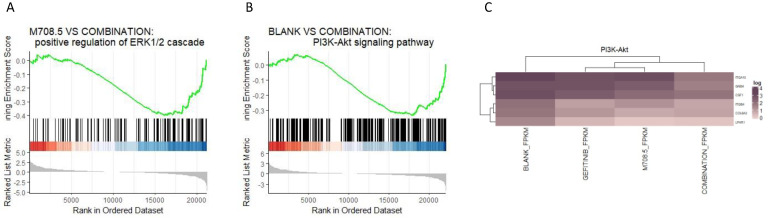
** The transcriptome analysis of the three conditions. A**. Gene set enrichment analysis (GSEA) of genesets for the comparison between m708.5 and combination conditions. **B**. GSEA of genesets for the comparison between blank condition and combination treatment. **C**. The heatmap of significantly differential expressed genes (DEGs) in the PI3K-Akt signaling pathway (adjusted P-value < 0.1).

We generated the bulk RNA-seq for the three conditions of the non-treatment, gefitinib treatment, combination treatment of m708.5 plus gefitinib. The gene set enrichment analysis (GSEA) indicated the ERK1 and ERK2 cascade process was significantly up-regulated in the combination of m708.5 and gefitinib compared with m708.5 only (*P-value* = 0.0016) (**Fig. 5A**). Moreover, GSEA suggested the up-regulation of PI3K-Akt signaling pathway in the comparison of the combination vs. the blank treatment (*P-value* = 0.0012) (**Fig. 5B, C**).

## Discussion

EGFR as a receptor tyrosine kinase involves in cell proliferation, motility, survival, and invasive ability. It was frequently expressed at relatively high density in adult and pediatric tumors 30, 31. Although EGFR was deemed a promising target for cancer therapy with significant clinical success in several epithelial cancers, outcomes of EGFR inhibitors including gefitinib are often unsatisfied in some patients 32. Gefitinib as a single agent has shown limited activity in neuroblastoma 33. Drugs tolerance is the basis for acquired resistance to gefitinib 34. Different molecular mechanisms play a role in resistance to EGFR inhibitors. The solo treatment of EGFR inhibitors is probably not sufficient to suppress tumor growth, calling for alternative therapy to solve drug resistance.

IGF-1R plays a crucial role in the emergence of cells tolerant to EGFR inhibitors. The IGF-1R axis is highly associated with multiple growth factors, receptors and downstream effectors. IGF-1R seems to be activated by crosstalk with EGFR on multiple levels, either through a direct receptor association or the shared downstream signaling molecules 35. It has been recently reported that crosstalk of IR/IGF1R and EGFR pathways results in reciprocal compensatory mechanisms that confers acquired resistance to individual treatment 29. A study demonstrates that the combination of EGFR inhibitor erlotinib and IGF-1R inhibitor NVP-AEW541 shows synergistic inhibition than either agent alone in adrenocortical carcinoma 36. This crosstalk of EGFR and IGF-1R provides a rationale for the dual targeting of EGFR and IGF-1R.

The IGF-I/II-neutralizing antibody m708.5 exhibited extremely high affinity to human IGF-I (*K*_D_ = 15 pmol/L) and to IGF-II (*K*_D_ = 9 pmol/L) as well as showed the significant anticancer activity in different human tumor cell lines through neutralizing IGFs 21. In this study, antitumor efficacy of m708.5 was further evaluated against neuroblastoma and breast cancer cells in combination with several chemodrugs, including EGFR inhibitor gefitinib *in vitro* and *in vivo.* The present results indicate that either gefitinib or m708.5 inhibits the cell growth of neuroblastoma (LAN-1) and breast cancer (MCF-7 and SK-BR-3) cell lines. Besides, our results further suggest that m708.5 has a very strong synergistic effect with gefitinib and synergism with cisplatin and pimasertib. Importantly, the combination of m708.5 IgG1 and gefitinib significantly results in much better tumor inhibition than that observed for either single treatment *in vivo*. In conclusion, m708.5 a promising therapeutic candidate shows high preclinical activity against neuroblastoma and breast cancer cells. Given the potent activity of IGF-I/II-neutralizing antibody when combined with EGFR inhibitors, both pathways commonly used by these tumors, such a combination may be clinically beneficial in the therapy of solid tumors.

## Figures and Tables

**Table 1:** * In vitro* growth inhibition by m708.5 in combination with drugs^a^

Cell line	gefitinib			pimasertib			cisplatin		
	Concentration	CI	Effect^b^	Concentration	CI	Effect^b^	Concentration	CI	Effect^b^
LAN-1	0.04-33.3μmol/L	0.012	very strong synergism	0.13-33.3μmol/L	0.002	very strong synergism	NA	NA	NA
MCF-7	0.04-33.3μmol/L	0.056	very strong synergism	NA	NA	NA	0.04-33.3μmol/L	0.03	very strong synergism
SK-BR-3	0.04-33.3μmol/L	0.14	strong synergism	0.41-33.3μmol/L	0.19	strong synergism	NA	NA	NA

^a^Concentration of m708.5 was fixed at 0.3-0.6 μg/mL. ^b^definition of CI values, <0.1 (very strong synergism); 0.1-0.3 (strong synergism); 0.3-0.7 (synergism); 0.7-0.85 (modest synergism); 0.85-0.9 (slight synergism); 0.9-1.1 (nearly additive); 1.1-1.2 (slight antagonism); 1.2-1.45 (modest antagonism); 1.45-3.3 (antagonism); 3.3-10 (strong antagonism); >10 (very strong antagonism).
